# Breastfeeding and swallowing in a neonate with mild hypoxic-ischaemic encephalopathy

**DOI:** 10.4102/sajcd.v64i1.209

**Published:** 2017-05-22

**Authors:** Esedra Krüger, Alta Kritzinger, Lidia Pottas

**Affiliations:** 1 Department of Speech-Language Pathology and Audiology, University of Pretoria, South Africa

## Abstract

**Background:**

Specific breastfeeding and swallowing characteristics in neonates with hypoxic-ischaemic encephalopathy (HIE) have not yet been well described in the literature. Considering the relatively high incidence of HIE in resource-poor settings, speech-language therapists should be cognisant of the feeding difficulties in this population during breastfeeding.

**Objective:**

To systematically describe the breastfeeding and swallowing of a single case of a neonate diagnosed with mild HIE from admission to discharge.

**Method:**

A case study of a 2-day old neonate with mild HIE in a neonatal intensive care unit at an urban teaching hospital, is presented. Data were prospectively collected during four sessions in a 12-day period until the participant’s discharge. Feeding and swallowing were assessed clinically, as well as instrumentally using a video-fluoroscopic swallow study.

**Results:**

After parenteral feeding, nasogastric tube feeding commenced. Breastfeeding was introduced on Day 6, as it was considered a safe option, and revealed problematic rooting, shallow latching, short sucking bursts, infrequent swallowing, and a drowsy state of arousal, with coughing and choking. No penetration or aspiration was identified instrumentally. After 13 days, the neonate was breastfeeding safely.

**Conclusion:**

Although the pharyngeal stage of swallowing was intact, symptoms of oral stage dysphagia were revealed using a combination of clinical and instrumental measures. Breastfeeding difficulties were identified, exacerbated by poor state regulation, which lead to prolonged hospitalisation. The case study highlights the unexpected long duration of feeding difficulties in an infant with mild HIE and indicates further research.

## Introduction

Hypoxic-ischaemic encephalopathy (HIE) describes abnormal neurologic behaviour in the neonate because of a hypoxic-ischaemic event (Groenendaal & De Vries, 2015). Organ dysfunction, specifically of the heart, lungs, gut, kidneys and the brain, is associated with HIE (Thyagarajan et al., 2015). HIE is referred to as a possible neurological cause of dysphagia (Dodrill & Gosa, 2015; Garg, 2004; Rybak, 2015). According to Sarnat and Sarnat observations (1976), as well as other HIE grading systems such as the HIE score (Thompson, Van der Elst, Molteno & Malan, 1997) or the modified Sarnat encephalopathy grading (Horn, 2013; Shalak, Laptook, Velaphi & Perlman, 2003), infants with HIE may be at risk of early feeding problems. This may be because of a weak or absent rooting and sucking reflex, which is distinctive of all three HIE stages [mild (I), moderate (II) and severe (III)] (Horn, 2013; Shalak et al., 2003).

Infants with neurological conditions may have significant feeding difficulties (Genna, LeVan Fram & Sandora, 2013). According to Tamminen, Verronen, Saarikoski, Göransson and Tuomiranta (1983), infants with neonatal encephalopathy because of hypoxia-ischemia, or birth asphyxia as it was previously named, may show breastfeeding difficulties. Inadequate muscular control in the infant’s oral area may be related to neurological deficits such as asphyxia (Wolf & Glass, 1992). A condition such as HIE may also impact the infant’s ability to achieve a correct latch-on for breastfeeding (Walker, 2017). Neurologic impairment in infants may furthermore inhibit state regulation, which may negatively influence an infant’s ability to feed successfully (Wolf & Glass, 1992).

Furthermore, gastrointestinal tract impairment, and specifically necrotising enterocolitis (NEC), is also a concern following HIE (Thyagarajan et al., 2015), which may further complicate feeding in this population. Infants with neurological impairment are likely to be more disorganised than neuro-typical infants as they are influenced by hospital procedures, medication, and are often separated from their mothers, which may affect their ability to breastfeed (Genna et al., 2013). Central nervous system involvement such as HIE is considered to be a known aetiology of dysphagia in infants (Arvedson & Brodsky, 2002), but globally, literature describing the specific early breastfeeding and swallowing characteristics in neonates with HIE is scarce.

Limited published data exist on the incidence of HIE in Sub-Saharan Africa (Horn, 2013). HIE has a range of reported incidences, but occurs approximately 1–5 per 1000 live births according to three studies in the United Kingdom, Australia and Sweden (Horn, 2013). Research in South Africa reported incidences of 8.3/1000 and 3.6/1000 (Bruckmann & Velaphi, 2015; Horn, 2013).

Because of the relatively high incidence of HIE in South Africa, speech-language therapists (SLTs) will encounter infants with HIE in their caseloads and will be required to be skilled in addressing feeding difficulties that may arise in this population. Infants with neurological conditions such as HIE may have various issues with feeding including muscle tone disturbances, inadequate coordination, hampered function of certain nerves or neuromuscular units, inappropriate state regulation and limited endurance for feeding (Genna et al., 2013). These challenges may influence early breastfeeding, but an infant may adapt over time, depending on the severity of the neurological condition (Walker, 2017).

The benefits of exclusive breastfeeding for infants have been documented well and are recommended for the first 6 months of life in high-income as well as low- to middle-income countries for all infants (Kramer & Kakuma, 2001; Victoria et al., 2016). Breastfeeding challenges that may arise from infants with neurological involvement may include sucking and swallowing difficulties, poor weight gain, separation from the mother in the first week of life, as well as maternal grief and shock at having an infant with a disorder (Walker, 2017).

The SLT being a central team member of an in-patient feeding and swallowing team should address the mentioned issues in affected families (Arvedson & Brodsky, 2002; Walker, 2017). SLTs, therefore, require an understanding of the profile of the feeding characteristics of neonates with HIE in order to render comprehensive services to this population and their families. The following research question was, therefore, posed: What are the early breastfeeding and swallowing characteristics including the feeding difficulties of a neonate with HIE in neonatal care? A description of the breastfeeding and swallowing characteristics in a case of HIE in a neonate over a period of 12 days is presented. A clinical description may contribute to the dearth of research on the early oral feeding attempts in infants with HIE in South Africa and abroad. The findings may also assist SLTs to identify the feeding difficulties in neonates with HIE that may lead to longer hospitalisation.

## Research method and design

### Aim

The aim of this study was to systematically describe the breastfeeding and swallowing of a neonate diagnosed with HIE over a period of 12 days until discharge from the hospital.

### Design

A case study was used, and data were collected prospectively. An in-depth description of a single case over time is presented (Leedy & Ormrod, 2014).

### Setting

Data were collected in the Neonatal Intensive Care Unit (NICU) and high care unit of an urban tertiary hospital in Pretoria. Neonates diagnosed with HIE and deemed suitable candidates, receive whole body therapeutic hypothermia for 72 h. Intermittent kangaroo mother care is provided after completion of therapeutic hypothermia. Neonates in the NICU and high care unit are provided with neurodevelopmental supportive positioning on warm tables and incubators. The hospital encourages exclusive breastfeeding and expressed breast milk (EBM) by cup as an alternative.

### Participant

An inborn post-term male neonate of 2 days old, diagnosed with HIE by a paediatrician, and receiving therapeutic hypothermia, was purposefully selected for this study. Characteristics of the infant and mother are shown in Table 1.

**TABLE 1:** Maternal and infant characteristics.

Characteristic	Category	Variable
Maternal	Age	41 years
	Antenatal visits	1
	Medication	HAART
Infant	Birth order	4th
	Birth weight	3.815 kg
	Gestational age using New Ballard score (Ballard et al., 1991)	42 weeks
	Mode of delivery	Emergency caesarean section
	Apgar 1 min	2/10
	Apgar 5 min	6/10
	Apgar 10 min	9/10
	Highest HIE score (Thompson et al., 1997)	4
	HIE stage (Sarnat & Sarnat, 1976)	Mild – stage 1
	Therapeutic hypothermia duration	72 h
	Supplementary ventilation	CPAP
	Duration	1 day
	Medication	Nevirapine; Antibiotic treatment (Amikacin, Penicillin)
	Other diagnoses	HIV exposure; Septicaemia

CPAP, continuous positive airway pressure; HAART, highly active antiretroviral treatment; HIE, hypoxic-ischaemic encephalopathy.

According to Table 1, the neonate’s highest HIE score (Thompson et al., 1997) was 4, indicating mild HIE, on Day 1. No congenital anomalies or metabolic disorders were diagnosed. Therapeutic hypothermia is the standard of care for infants with HIE globally (Tagin, Woolcott, Vincer, Whyte & Stinson, 2012). According to the literature, an infant is diagnosed with HIE by a medical doctor when the following criteria are met (Martinez-Biarge, Diez-Sebastian, Wusthoff, Mercuri & Cowan 2013; Volpe, 2012): arterial cord blood gas indicative of metabolic acidosis (pH < 7.1), depressed Apgar scores (<7 at 5 and/or 10 min), the presence of neonatal encephalopathy (depression of level of consciousness, usually with respiratory difficulty, abnormal muscle tone, disturbed cranial nerve function especially impaired feeding and often the presence of seizures) and the need for resuscitation.

As further indicated in Table 1, the neonate was exposed to HIV and received Nevirapine, but it was too early to suspect the presence of HIV encephalopathy. The neonate’s mother was of advanced age and received limited antenatal care in comparison with the minimum of four antenatal appointments prescribed for mothers with HIV (National Department of Health, 2015). Mothers who are HIV positive in this hospital setting are counselled about exclusive breastfeeding during antenatal care and at birth as per National Department of Health (2015) guidelines. Both mother and infant receive antiretroviral treatment, and viral loads are monitored to ensure safe breastfeeding.

### Materials

Well-known literature on paediatric dysphagia and standard parameters of feeding such as coordination of sucking, swallowing and breathing, respiration during feeding and swallowing (Arvedson & Brodsky, 2002; Pereira, Sacher, Ryan & Hayward, 2009; Wolf & Glass, 1992) were used to compile a data collection sheet and to select relevant information from the hospital file. Certain components of the oral motor and feeding assessment checklist (Arvedson & Brodsky, 2002) were extrapolated for the data collection sheet. This checklist obtains relevant history of the infant and mother, includes a pre-feeding observation of the infant at rest, including a physical examination (such as muscle tone and respiration), as well as observations of non-nutritive sucking and a feeding session at the breast. The HIE score (Thompson et al., 1997), calculated by the paediatrician, was obtained from the hospital file. The Preterm Infant Breastfeeding Behavior Scale (PIBBS) (Nyqvist, Rubertsson, Ewald & Sjöden, 1996) was used to monitor the progress of breastfeeding. The PIBBS may be used with full-term infants and was designed as a breastfeeding monitoring tool for use by mothers or clinicians (Da Costa, Van den Engel-Hoek & Bos, 2008; Nyqvist, 2013).

A video-fluoroscopic swallow study (VFSS) was performed using a SYSCO 19’ version Multi DiagnostEleva FD (Philips, Netherlands) screening machine. The contrast liquid used during the VFSS was a commercially prepared non-ionic X-ray contrast solution. The contrast liquid was presented in a NUK™ bottle using a narrow NUK™ teat (birth to 6 months) with a small hole. A data collection sheet was compiled for the interpretation of the VFSS, which included a checklist compiled from Arvedson and Brodsky (2002), as well as the Penetration Aspiration scale (PAS) (Rosenbek, Robbins, Roecker, Coyle & Wood, 1996). The PAS is an eight-point classification system to quantify the penetration and aspiration events during VFSS (Rosenbek et al., 1996). The PAS was conceptualised for use with adults, and thus far, data are only available for use in the adult population (Arvedson & Brodsky, 2002), but has been used in a study on the paediatric population (Masarei, Wade, Stat, Mars & Sommerlad, 2007).

### Procedure

The participant’s feeding was clinically evaluated daily by the first author (Esedra Krüger), an SLT registered with the Health Professions Council of South Africa (HPCSA), experienced in assessment of feeding and swallowing in neonates. The assessments were completed during typical feeding sessions in the NICU once the participant was deemed ready for oral feeding by the managing paediatrician. Multiple data collection entries were made to allow for capturing of follow-up data. Information on the neonate’s medical treatment from admission, as well as his progress, was obtained from the hospital file. The neonate was treated daily, by providing parent guidance and oral stimulation, by a hospital-based SLT (Arvedson & Brodsky, 2002).

The PIBBS (Nyqvist et al., 1996) was completed daily from the commencement of oral feeding on Day 6, during direct observation of breastfeeding. Direct observation is the least intrusive method of observing breastfeeding while not disrupting the infant’s behaviour (Nyqvist et al., 1996). The researcher stood near the mother in a position that provided a clear view of the neonate’s face and chin, according to the description by Nyqvist et al. (1996).

The neonate was instrumentally assessed on Day 13 using VFSS, according to recommendations by Arvedson and Brodsky (2002). The lateral projection view was used, as it yields the most valuable information when studying the paediatric population (Arvedson & Brodsky, 2002; DeMatteo, Matovic & Hjartarson, 2005). The VFSS was conducted by two hospital-based SLTs registered with the HPCSA who has experience in VFSS in infants, in conjunction with a radiologist and a radiographer based at the hospital.

### Analysis

Clinical assessment data were systematically collected by the researcher via observation, without being involved in treatment of the participant (Meline, 2010). The clinical assessment data were interpreted using normative data available for full-term infants (Arvedson & Brodsky, 2002; Walker, 2017). The VFSS recording, which allowed a frame by frame analysis, was analysed by a panel of two SLTs experienced in interpretation of VFSS in infants. The panel was blinded to the results of the clinical feeding assessments conducted by the researcher.

## Results

**FIGURE 1:**
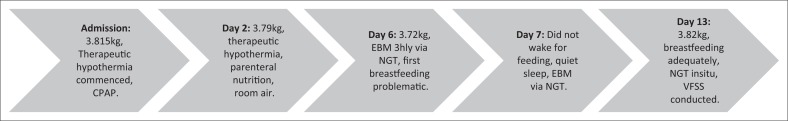
Chronological events from admission to discharge.

The results are described chronologically according to the occurrence of events in the participant’s 14-day stay in the hospital and are depicted in Figure 1.

Rooting and sucking reflexes were reported to be absent by the paediatrician upon the participant’s admission to the NICU. During the initial clinical assessment on Day 2, with a weight of 3.79 kg, the neonate was still receiving whole body therapeutic hypothermia (Figure 1). At the scheduled feeding time, he was lethargic, and difficult to rouse, presented with hypotonia, and was unresponsive to stroking on and around the mouth. Non-nutritive sucking could not be elicited, although minimal jaw and tongue movement were perceived. No EBM was available yet, and the participant received only parenteral nutrition.

The participant received EBM 3-hourly via nasogastric tube (NGT) on the following day after therapeutic hypothermia (Day 3). On Day 6, the participant was breathing room air, saturating well (>95%) (Wolf & Glass, 1992) and weighing 3.72 kg. The participant was physiologically stable, with his condition improving, while being cared for in the high care unit positioned in side-lying in an open bassinet. He was deemed ready to commence oral feeding by the paediatrician and the hospital-based SLT. The participant was positioned cradled in his mother’s arm for all breastfeeding attempts, which is a comfortable position for most feeders (Wolf & Glass, 1992). Communication interaction between the mother and the participant during all feeding sessions could be described as inadequate with limited eye contact. The results of the PIBBS are presented in Table 2.

**TABLE 2:** Breastfeeding results according to the Preterm Infant Breastfeeding Behavior Scale.

PIBBS	Day 6	Day 7	Day 13
Rooting	Some	None	Obvious
Latching on breast	Only nipple	Nothing, only touched nipple	Whole nipple, part areola
Duration of latching (min)	<1	0	11
Duration of breastfeeding session (min)	41	45	15
Sucking	Single sucks, occasional short bursts (2–4)	None	Repeated long bursts (>10)
Longest sucking burst	4	0	>30
Swallowing	Occasional	None	Repeated
Behaviour	Drowsy, minimum movements, eyes open but heavy-lidded	Closed eyes, no body movement	Eyes wide open, eye contact, calm, minimum movements
Let-down reflex	Noticed	Not noticed	Noticed
Breast problems	None	None	None
Environment	Activity nearby, rather disturbing	Activity nearby, rather disturbing	Activity nearby, rather disturbing

PIBBS, Preterm Infant Breastfeeding Behavior Scale.

On Day 6, the participant displayed some rooting, but showed shallow latching onto only the nipple, and stayed latched onto the breast for less than 1 min (see Table 2). Single sucks and occasional short sucking bursts of two to four sucks were observed. Occasional swallowing was noticed, but coughing and choking were present. The participant was predominantly in a drowsy state before and during feeding. Premature fatiguing, as well as hiccupping and yawning, were observed. The breastfeeding session lasted approximately 41 min, and the infant received EBM via the NGT to complete the feed. EBM via cup feeding was attempted by hospital staff when his mother was not available to breastfeed. The infant received intervention by the SLT daily. Information available from the participant’s hospital records indicated that cup feeding was successful, however, the daily SLT intervention records indicated otherwise.

The participant was in the quiet-sleep stage at the scheduled feeding time on Day 7. He presented with closed eyes and showed no body movements even with repeated attempts at waking him for the feeding. No rooting or latching could be elicited during breastfeeding, and no swallowing was noticed. NGT feeding continued supplemental to breastfeeding, and a VFSS was scheduled for the following week.

During the VFSS on Day 13, no penetration or aspiration was identified. No residue was left on the posterior pharyngeal wall, the valleculae or the pyriform sinuses. However, the qualitative analysis revealed poor bolus formation and delayed oral transit time, which may be indicative of oral stage dysphagia (Arvedson & Brodsky, 2002).

On Day 13, the participant had regained his birth weight at 3.82 kg and was mainly breastfed, although a NGT was still *in situ*. The participant was awake and alert before and during feeding. He showed obvious rooting, latched deeply onto the whole nipple and some of the areola, and stayed latched onto the breast for approximately 11 min. Repeated long sucking bursts (>10 sucks per burst), and repeated swallowing, indicative of successful feeding, were observed. The participant was discharged home the following day. The participant and his mother continued with antiretroviral treatment.

### Validity and reliability

The outcome measures used in the study are published and deemed valid and reliable tools to use for the assessment of infant feeding. The reliability and validity of the PIBBS was considered satisfactory and increased the rigour of this study (Da Costa et al., 2008; Nyqvist et al., 1996). The VFSS is referred to as the ‘gold standard’ for examining swallowing (Arvedson & Brodsky, 2002), which contributed to the validity of the results. The use of two blinded raters for the VFSS increased the reliability of the VFSS findings.

## Ethical considerations

Ethical clearance was obtained from the hospital, as well as from the Faculties of Health Sciences and Humanities of the University of Pretoria prior to data collection. Voluntary informed consent was requested from the mother on behalf of her infant to participate in the study and for the medical file to be perused. The mother was informed that she may withdraw her infant from the study at any time without her usual care at the hospital being affected. The participant received intervention from a hospital-based speech-language therapist from the day of the first assessment. Data were handled confidentially and will be stored electronically and in hard copy according to institutional guidelines for a period of 15 years.

## Discussion

A neonate with mild HIE and HIV exposure, as well as feeding difficulties, was presented. Weight loss was minimal, and the participant regained his birth weight at the last assessment prior to discharge. Problems with rooting and latching onto the breast were identified while the participant was treated for septicaemia and after receiving therapeutic hypothermia and continuous positive airway pressure (CPAP) in the NICU.

The infant presented with hypotonia initially. Muscle tone problems may lead to incorrect alignment, which may contribute to feeding difficulties (Wolf & Glass, 1992). The participant received parenteral and tube feeding initially. Feeding difficulties requiring tube or intravenous feeding are signs of cerebral depression that may likely correlate with future neurological dysfunction (Genna et al., 2013). Successful latching-on and breastfeeding require an intact central nervous system (Radzyminski, 2005).

Sucking and swallowing were not optimal for breastfeeding between birth and Day 13. He was hospitalised for 14 days, mainly because of an inability to complete all feeds orally, and possibly because of reduced endurance (short sucking bursts and staying on the breast less than a minute). Breastfeeding sessions may have been prolonged as the mother was motivated for her son to breastfeed and was able to dedicate time to breastfeeding. The required daily volume of feeds per day was ensured with NGT feeding. Successful breastfeeding was not established until the second week after birth. Infants with neonatal encephalopathy because of hypoxia-ischemia may show difficulty starting to breastfeed (Tamminen et al., 1983). The participant was his mother’s fourth child, and her advanced age and prior child-rearing experience may have contributed to successful feeding after 13 days. Evidence also exists that an infant’s ability to increase his or her intake at the breast develops over time (Nyqvist, 2001; Walker, 2017).

Although no clinical symptoms of oral stage dysphagia, associated with HIV, were observed, the participant’s HIV exposure may have contributed to problematic feeding. HIV exposure in itself is linked with subtle neurodevelopmental difficulties (Le Doaré, Bland & Newell, 2012), which may likely influence feeding in a young infant. A neonate with mild HIE may present with absent rooting and sucking reflexes initially (Thompson et al., 1997), which was true in this case. However, rooting and sucking remained inadequate for breastfeeding well into the first week of the participant’s life. An absent rooting reflex may not impact an infant’s feeding functionally, but short sucking bursts may be related to swallowing difficulties (Wolf & Glass, 1992).

The participant’s state was drowsy before and during feeding. Quiet-alert, drowsy or active alert may be the optimal states to feed a neonate (Arvedson & Brodsky, 2002). Although the drowsy state is labelled as being one of the optimal states for feeding, there may only be one specific state for a particular infant that is ideal for feeding (Wolf & Glass, 1992). State is also related to an infant’s neurological and medical status (Wolf & Glass, 1992). Cerebral depression may cause reduced alertness and a lower level of consciousness in infants (Genna et al., 2013). In this study, it appears that state regulation may have affected the participant’s ability to feed successfully.

The instrumental assessment of swallowing revealed no aspiration, penetration or residue. However, clinical signs of aspiration, such as coughing and choking, which may also be signs of stress (Arvedson & Brodsky, 2002), were observed on Day 6, while the VFSS was only conducted on Day 13. The participant received intervention from the SLT during this period, which may have contributed to the improvement in the coordination of the oral and pharyngeal stages of the swallow seen on the VFSS. Further research is required.

The VFSS during the second week revealed mild symptoms of oral stage dysphagia, which is similar to the findings in the clinical assessment of breastfeeding. From this case presentation, it is clear that feeding in a neonate with HIE and HIV exposure, was not only affected because of the absence of rooting and sucking reflexes, but also because of the neonate’s inability to maintain an appropriate state for feeding. The participant’s state may have been influenced by the diagnosis of HIE, and his medical status, namely the provision of CPAP and therapeutic hypothermia in the first days of life as well as treatment of septicaemia (Wolf & Glass, 1992), and the fact that he was a post-term infant, or even a combination of these factors. Post-term infants (>42 weeks gestation) may often be lethargic and may have difficulty sustaining sucking (Walker, 2017).

Because of a relatively high incidence of HIE in South Africa, SLTs working in hospitals are required to provide the earliest feeding intervention to this population of infants. This case study outlines early salient features of the breastfeeding and swallowing of a neonate with HIE with HIV exposure. Although HIE is not a new diagnosis in an SLTs case load, this case study provides a novel description of the unique feeding profile that may be found in infants with HIE, namely, difficulty establishing breastfeeding initially because of poor latching, inadequate sucking and mild oral stage dysphagia. These difficulties coupled with HIV exposure and inadequate state regulation may have led to a longer hospitalisation and separation from the participant’s mother. As oral motor control, muscle tone and the infant’s state are interrelated, a deviation in any of these areas will likely influence the others and may lead to dysfunction in feeding (Wolf & Glass, 1992). Furthermore, separation of an infant and mother leads to increased stress in both and negatively influences breastfeeding (Smith, 2013).

In many instances, infants with mild HIE may escape the attention of the SLT with a large caseload, unlike the moderate or severely involved infant with HIE. The long duration of feeding difficulties after the initial recovery of mild HIE in the participant was unexpected. This case study highlighted the fact that feeding difficulties and prolonged hospital stay may be the result in a case of mild HIE and HIV exposure, and should, therefore, form part of the SLTs caseload. According to Gagne-Loranger, Sheppard, Ali, Saint-Martin and Wintermark (2016), newborns with mild neonatal encephalopathy may require more thorough monitoring as their outcomes have been less well studied.

The fact that the present study describes only one case, limits the generalisation of the findings to other infants with HIE. The study presented in-depth data on one case, which may point to the need to investigate the population of infants with HIE as a group. A larger scale investigation of the feeding characteristics of infants with HIE may therefore be valuable in order to assess the impact of HIE on infants’ initial feeding attempts, feeding practices used during therapeutic hypothermia, as well as neonatal communication development of this population.

The influence of HIV exposure on neonates’ early feeding skills also requires further research, as this case study cannot provide clarity on the influence of the participant’s HIV exposure on the resultant feeding outcomes of the participant. Because of the design of this study, it is also not clear what the influence of intervention by the hospital-based SLT was on the participant’s outcome. Future research may be directed towards the feeding intervention and neuro-developmental supportive care infants with HIE receive in neonatal care. The findings of this study may lead to SLTs’ improved understanding of the complexity of the feeding of even the mildly affected infants with HIE who also have HIV exposure.

## Conclusion

The specific breastfeeding difficulties, oral stage dysphagia and inadequate state regulation in this case study of a neonate with mild HIE and HIV exposure were presented in order to contribute to data on feeding characteristics of infants with HIE. Even though being diagnosed as having mild HIE, the participant’s problematic feeding led to prolonged hospitalisation. Neonates with HIE and HIV exposure may have unique feeding profiles. The early involvement of the SLT in the treatment of feeding difficulties in infants with mild HIE was highlighted. Further research investigating the feeding and communication development of infants with HIE is required.
